# Brief Submotor-Threshold Electrical Stimulation Applied Synchronously Over Wrist Flexor and Extensor Muscles does Not Suppress Essential Tremor, Independent of Stimulation Frequency

**DOI:** 10.5334/tohm.740

**Published:** 2023-09-01

**Authors:** Christian Metzner, Adam Stringham, Brenna Hislop, Joseph Bonham, Larrisa Chatterton, Ryan DeFigueiredo, Steven K. Charles

**Affiliations:** 1 Neuroscience, Brigham Young University, Provo, UT, USA; 2 Mechanical Engineering, Brigham Young University, Provo, UT, USA

**Keywords:** tremor suppression, sensory stimulation, essential tremor, synchronous stimulation, hand acceleration, surface electromyography

## Abstract

**Background:**

Electrical stimulation of muscles below motoneuron threshold has shown potential as a low-cost and minimally invasive treatment for Essential Tremor (ET). Prior studies have stimulated wrist flexor and extensor muscles synchronously with diverging results, calling for further investigation. Also, prior studies have only used a narrow range of stimulation parameters, so stimulation parameters have not been optimized. Our purpose was to further investigate synchronous submotor stimulation and identify the effect of stimulation frequency on tremor suppression.

**Methods:**

We quantified the effect of brief, synchronous stimulation at 15 different frequencies from 10–150 Hz applied over wrist flexors and extensors on both tremor power and frequency in 20 ET patients. We compared tremor power and frequency from hand acceleration and sEMG between pre-, per-, and post-stimulation phases.

**Results:**

Our stimulation paradigm did not result in significant tremor suppression or tremor frequency changes at any tested stimulation frequency, showing no significant interaction between phase and stimulation frequency for tremor power measured by either hand acceleration (p = 0.69) or sEMG (p = 0.07). Additionally, the effect of phase interacting with stimulation frequency on tremor frequency was statistically insignificant for acceleration (p = 0.64) and sEMG (p = 0.37).

**Discussion:**

We conclude that brief synchronous submotor-threshold stimulation does not reduce tremor in ET patients, independent of stimulation frequency (from 10 to 150 Hz). Our results are consistent with the hypothesis that brief submotor-threshold stimulation suppresses tremor via reciprocal inhibition, which requires asynchronous stimulation. In contrast, it is hypothesized that synchronous stimulation might require longer stimulation durations to affect supraspinal tremor networks.

**Highlights:**

We studied the effects of synchronous submotor electrical stimulation over wrist flexor and extensor muscles on Essential Tremor. Our results indicate that suppressing tremor with brief synchronous stimulation is ineffective. Based on recently hypothesized mechanisms of peripheral tremor suppression, we hypothesize that asynchronous stimulation or long-duration synchronous stimulation are more effective approaches to peripheral tremor suppression.

## Introduction

Essential Tremor (ET), which is characterized by postural and kinetic tremor in the upper limbs, is the most common movement disorder, affecting an estimated 7 million people in the United States [1,2]. Unfortunately, the two most common treatment methods, medication and deep brain stimulation (DBS), provide only partial relief. Medication is most popular, although only roughly 50% of patients see a reduction in tremor, with those patients showing only a 50% improvement [1]. In addition, patients commonly experience side effects such as headaches, dizziness, and nausea, as well as needing to increase dosage as they adapt to the medicine. Patients who have undergone unilateral deep brain stimulation (DBS) show 60% to 90% tremor reductions, but only 1 in 30 ET patients undergoes DBS surgery [3], likely in part because of the procedure’s invasiveness. Consequently, many patients are left without satisfactory treatment options [4].

Electrical stimulation of peripheral limbs to suppress tremor has been suggested as a non-invasive alternative to medication and DBS [5,6]. Over the years, different stimulation approaches have been developed (Table 1). In terms of intensity, there are two basic approaches: supramotor stimulation (also called functional electrical stimulation) and submotor stimulation (also called sensory stimulation). Supramotor stimulation uses stimulation intensities above motoneuron activation threshold, directly activating muscles. Submotor stimulation, on the other hand, stimulates below motoneuron activation threshold but above afferent neuron activation threshold, thus predominantly recruiting Ia proprioceptive afferents and cutaneous sensory afferents without directly activating muscle [7]. In addition to these two differing intensities, peripheral electrical stimulation can be divided into two timing strategies: asynchronous and synchronous stimulation. In asynchronous stimulation (sometimes called out-of-phase), antagonist muscle groups are stimulated out-of-phase with each other at the tremor frequency so that the stimulation-induced muscle responses (evoked by supramotor or submotor stimulation) cancel out tremor-induced muscle activity [8]. In synchronous stimulation (sometimes called continuous), muscles, including antagonist muscles, are stimulated simultaneously; synchronous supramotor stimulation results in co-contraction [8], whereas the exact mechanism of synchronous submotor stimulation is not well understood, although several hypotheses revolving around supraspinal effects have been proposed (see Discussion).

**Table 1:** Stimulation parameters used in previous studies. FCR, FCU, ECR represent flexor carpi radialis, flexor carpi ulnaris, and extensor carpi radialis muscles, respectively. ET stands for Essential Tremor, and NA stands for “not available”. Negative tremor change indicates tremor reduction during/following the stimulation.


FIRST AUTHOR	YEAR	STIMULATION PARAMETERS						NUMBER OF PATIENTS	TREMOR CHANGE

		MAGNITUDE	AGONIST-ANTAG. TIMING	FREQ (HZ)	PULSE WIDTH (µS)	DURATION (MIN)	SITE		

Javidan [10]	1992	Above motor threshold	Asynch.	30	100	NA	NA	24 (3 ET)	–73%

Maneski [11]	2011	Above motor threshold	Asynch.	40	250	Intervals of 0.05	Wrist extensors and flexors	7 (3 ET)	–67 ± 13%

Grimaldi [32]	2011	Above motor threshold	Synch.	30	100	0.33	Biceps, Triceps, FCR, ECR	3 (1 ET)	–50%

Widjaja [33]	2011	Above motor threshold	Asynch.	25	200	0.167 × 2	FCU and ECR	1 (1 ET)	–57%

Gallego [13]	2013	Above motor threshold	Synch.	30/40	250/300	0.25	FCU and ECR	6 (4 ET)	–52 ± 25%

Bo [12]	2014	Above motor threshold	Synch.	40	150	Intervals of 0.167–0.833	Wrist, fingers or thumb/index	10 (10 ET)	–60 ± 27%

Dosen [9]	2015	Above motor threshold	Asynch.	100	300	2	Wrist flexor and extensor muscles	6 (2 ET)	–60 ± 14%

		Below motor threshold	Asynch.	100	300	2	Wrist and finger flexor and extensor muscles	6 (2 ET)	–42 ± 5%

Heo [18]	2015	Below motor threshold	Synch.	100	300	NA	Elbow and wrist muscles	18 (all ET)	–90% (max. tremor reduction)

Heo [19]	2016	Below motor threshold	Synch.	100	300	NA	Elbow and wrist muscles	18 (all ET)	–12–24%

Dideriksen [14]	2017	Below motor threshold	Asynch.	100	400	0.5 × 2	Intramuscular	4 (2 ET)	–54 ± 20%

							Surface	5 (2 ET)	–50 ± 41%

Lin [16]	2018	Below motor threshold	Asynch.*	150	650	40	Median and Radial Nerves	10 (all ET)	–60 ± 8.4%

Pahwa [15]	2019	Below motor threshold	Asynch.*	150	650	40	Median and Radial Nerves	40 (all ET)	–59 ± 13%

Isaacson [17]	2020	Below motor threshold	Asynch.*	150	650	40	Median and Radial Nerves	205 (all ET)	–72%

Pascual-Valdunciel [20]	2021	Below motor threshold	Synch.	100	400	0.5	Surface	6 (all ET)	+30 ± 14%

					200	0.5	Intramuscular	7 (all ET)	+40 ± 40%

			Asynch.	100	400	0.5	Surface	8 (all ET)	–6 ± 16%

					200	0.5	Intramuscular	6 (all ET)	–26 ± 6%


* Unlike the other studies that used asynchronous stimulation, in these studies, stimulation was administered over median and radial nerves at the wrist joint (not antagonist muscles), and the timing of the stimulation was not synchronized with the patient’s tremorogenic activity or tremor.

Several studies have attempted supramotor peripheral stimulation for tremor suppression using both the asynchronous [9,10,11] and synchronous timing strategies [12,13]. While this stimulation intensity reduced tremor by up to 64%, patients experienced high levels of muscle fatigue and discomfort. Thus, most recent investigations of peripheral electrical stimulation for tremor suppression have focused on submotor stimulation.

Although the tremor-suppressing mechanism of submotor peripheral stimulation is only partially understood, it has shown potential in some studies (see an overview in Table 1), particularly for asynchronous administration. Dosen et al. administered brief asynchronous submotor stimulation over wrist and finger flexor and extensor muscles to five patients and found a decrease in tremor of 35–48% [9]. Similarly, Dideriksen et al. observed that brief asynchronous submotor stimulation of forearm muscles suppressed tremor by an average 52% for nine patients [14]. Using the Cala system, Pahwa et al. applied asynchronous stimulation to the median and radial nerves (at a constant frequency independent of the subject’s tremor unlike most asynchronous studies), resulting in an average postural tremor decrease of 59% in 40 ET patients, mirroring previous results seen by Lin et al [15,16]. These findings were confirmed in a large clinical study by Isaacson et al. showing a reduction in mean postural tremor power of 72% in 205 ET patients using the same stimulation paradigm and Cala system as Pahwa et al [17].

The studies using synchronous submotor stimulation have shown diverging results. Heo et al. found that brief, synchronous surface stimulation over wrist and elbow muscles in 18 patients decreased postural tremor by up to 90% during stimulation [18]. However, using the same method to reduce kinetic tremor, Heo et al. found only a 12% decrease during stimulation [19]. In contrast, Pascual-Valdunciel et al. found that brief, synchronous submotor intramuscular stimulation actually increased subjects’ tremor by up to 48% in the short term while brief, synchronous submotor surface stimulation had no significant effect on tremor power [20]. These diverging results call for further investigation of the synchronous stimulation strategy. In addition, the effect of stimulation parameters (frequency, amplitude, pulse width, duration, timing relative to tremor, etc.) on synchronous submotor peripheral stimulation have not been explored systematically. For example, the aforementioned studies stimulated at only one of two frequencies (either 100 Hz or 150 Hz), with little justification for either. Finally, while all the studies mentioned above investigate tremor power and severity, little is known about the effects of submotor stimulation on tremor frequency. Any shifts in tremor frequency during stimulation would indicate potential supraspinal mechanisms as suggested by studies connecting tremor frequency to central tremor generation [21].

To start addressing these gaps, we performed a systematic investigation of the effect of stimulation frequency (applied in synchronous submotor stimulation) on postural tremor power and frequency in ET. Thus, this study 1) sheds light on the diverging results of past synchronous submotor stimulation studies by systematically testing synchronous submotor stimulation across a large range of simulation frequencies, 2) tests the hypothesis that some stimulation frequencies might be more effective than others, and 3) examines the effect of synchronous submotor stimulation on tremor frequency as well as tremor power.

## Methods

### Subjects

Twenty patients with ET diagnosed by a neurologist were recruited to participate in our experiment (Table 2). All subjects were right-handed. 13 subjects were on medication at the time of the experiment. Each subject was evaluated with The Essential Tremor Rating Assessment Scale (TETRAS), using only the parts of the scale related to upper-limb tremor [22]. A numerical value of tremor severity was calculated by averaging the test scores evaluated in the upper limb with the more severe tremor. Following procedures approved by Brigham Young University’s Institutional Review Board, written informed consent was obtained from all subjects.

**Table 2:** Patient information. Patients are ordered from lowest to greatest TETRAS score.


PT #	SEX	AGE	AGE OF ONSET	TREMOR DURATION (YEARS)	WRIST TESTED	HT (CM)	WT (KG)	FAMILYHISTORY	TREMOR SEVERITY (TETRAS)*	ON MEDICATION

6	F	58	39	19	L	165	88	Yes	0.3	No

15	F	27	12	15	L	160	84	Yes	1.2	Yes

5	F	59	15	44	R	152	64	No	1.5	Yes

9	M	66	56	10	R	191	134	Yes	1.5	Yes

19	F	55	23	32	R	155	109	Yes	1.7	No

12	M	19	16	3	R	180	66	No	1.9	Yes

7	M	30	10	20	R	191	82	No	2.3	No

11	M	74	54	20	R	180	70	No	2.3	Yes

14	F	41	14	27	R	175	64	Yes	2.3	No

4	F	71	10	61	R	160	75	Yes	2.5	Yes

10	M	82	62	20	R	165	62	Yes	2.5	No

16	F	57	22	35	L	163	72	Yes	2.5	Yes

17	M	74	15	59	R	168	75	No	2.5	No

20	F	57	30	27	R	168	73	Yes	2.5	No

1	M	64	48	16	L	196	125	No	2.7	Yes

2	F	80	65	15	L	157	61	No	2.7	Yes

18	F	68	54	14	L	168	79	No	2.7	Yes

8	F	67	58	9	R	165	94	Yes	2.8	Yes

13	M	70	10	60	L	178	104	Yes	2.9	No

3	F	55	20	35	R	160	65	Yes	3.4	Yes


* Average of scores related to upper-limb tremor; scores of 2, 3, and 4 are associated with mild, moderate, and severe tremor, respectively.

### Experimental Preparation and Setup

#### Application of Electrodes and sEMG Sensors

**Figure 1:**
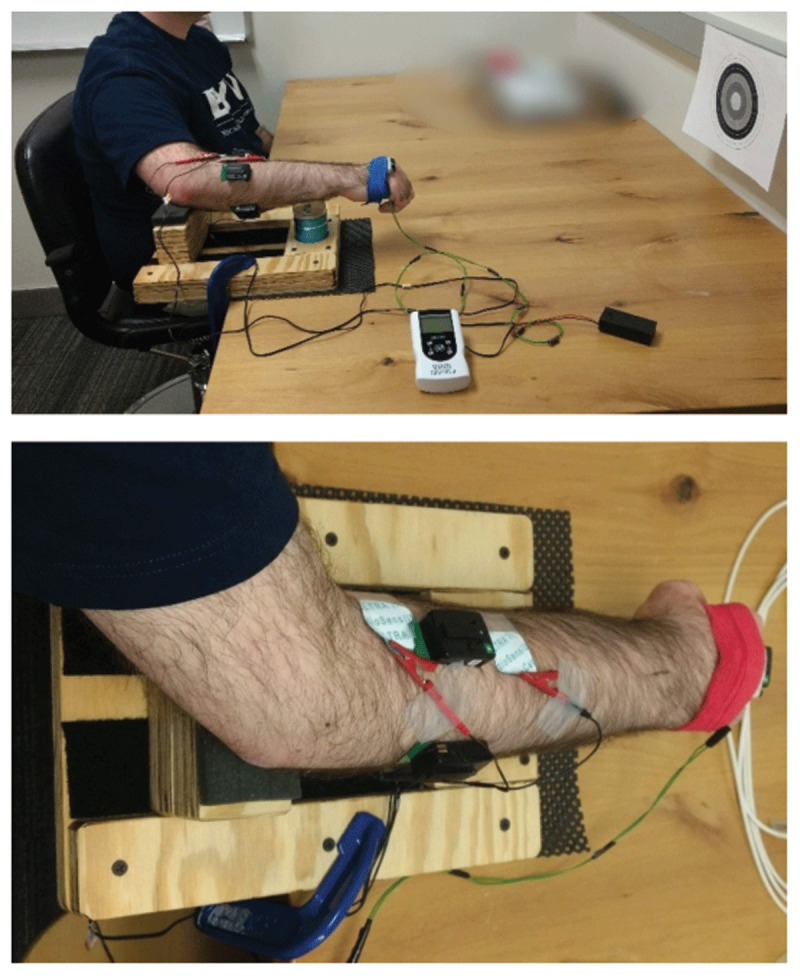
Experimental setup. Subjects rested their forearm on supports proximally and distally, allowing the hand to move freely. Subjects were instrumented with an inertial measurement unit and a laser pointer on the hand (under the blue/red wrap), TENS electrodes straddling the extensor carpi radialis longus and brevis (visible in lower photo) and flexor carpi radialis (not visible), and sEMG sensors over the extensor carpi radialis and ulnaris (visible in both photos) and over the flexor carpi radialis and ulnaris (not visible). Subjects were asked to point at the target in front of the subject (visible in top photo).

We used a TENS device (InTENSity TwinStimm III by Roscoe Medical, Strongville, OH) to provide submotor electrical stimulation to both the anterior and posterior sides of the forearm (Figure 1). Specifically, one pair of 1” × 1” electrodes (CardioSens/Ultra II, Mortara Instrument, Milwaukee, WI) was placed 3 inches apart over the belly of the flexor carpi radialis (FCR) muscle, and another pair was placed over the bellies of the extensor carpi radialis longus and brevis muscles (ECR; due to the proximity of these muscles, a single pair of electrodes was used for both). The electrodes and cable leads were secured to the subject’s arm with medical tape (Transpore by 3 M, Neuss, Germany). The TENS unit was set to 200 µs pulse width for all trials. In addition, surface electromyography (sEMG) sensors (Trigno EMG Sensors by Delsys Inc., Natick, MA) were placed over FCR, ECR, flexor carpi ulnaris (FCU), and extensor carpi ulnaris (ECU) muscles and secured using self-adherent wrap (Sensi-Wrap by Dynarex, Orangeburg, NY).

#### Measurement of Sensory and Motor Thresholds

Throughout the experiment, subjects were stimulated above sensory threshold (where stimulation is first felt) but below motor threshold (where stimulation first causes muscle contraction). This required finding sensory and motor thresholds for each subject at various stimulation frequencies. We measured each subject’s sensory and motor thresholds at 10, 50, 100, and 150 Hz, and then interpolated for stimulation frequencies in-between. Specifically, the TENS device was set at 100 Hz and current was increased from zero in increments of 1 mA (the smallest resolution available in the device) until the subject indicated that stimulation was felt (sensory threshold). The current was further increased until involuntary muscle contraction was detected (motor threshold), usually indicated by twitching fingers or pulsing muscles. This process was repeated for stimulation frequencies of 150, 50 and 10 Hz on both anterior and posterior sides of the arm. Once sensory and motor thresholds were measured, stimulation current was set halfway between sensory and motor thresholds (rounding up to the nearest integer mA). Stimulation amplitudes at other frequencies were found by interpolation.

Sensory threshold was detected on both flexor and extensor sides of the forearm in all patients. However, in 8 patients, we were unable to detect motor threshold on either one or both sides of the forearm despite raising the current as high as 16 mA. Two of these subjects had tremor so intense that it was difficult to distinguish stimulation-induced motion from tremor-related motion. When no motor threshold was found, we stimulated at the maximum comfortably tolerable current, which was always greater than the subject’s sensory threshold and, in almost all cases, greater than the average stimulation current and below the average motor threshold of the other subjects. To make sure these 8 subjects did not significantly influence our results, we ran our statistical analyses with and without them (see Limitations).

#### Setup

Subjects were seated next to a sturdy table (Figure 1). The arm showing the most tremor was used for testing (Table 2). Subjects sat with their shoulder abducted approximately 45 degrees and flexed approximately 30 degrees. The elbow was flexed approximately 45 degrees from full extension, and the forearm was pronated and rested comfortably on a support device clamped to the table. The support allowed free motion of the hand in both wrist flexion-extension and radial-ulnar deviation. A laser pointer and an inertial measurement unit (IMU) (Trigno IM Sensors by Delsys Inc., Natick, MA) were secured to the back of the subject’s hand with self-adherent wrap. A target was attached to the wall such that the laser pointer illuminated the target’s center when the subject’s wrist was approximately in neutral position.

**Figure 2:**
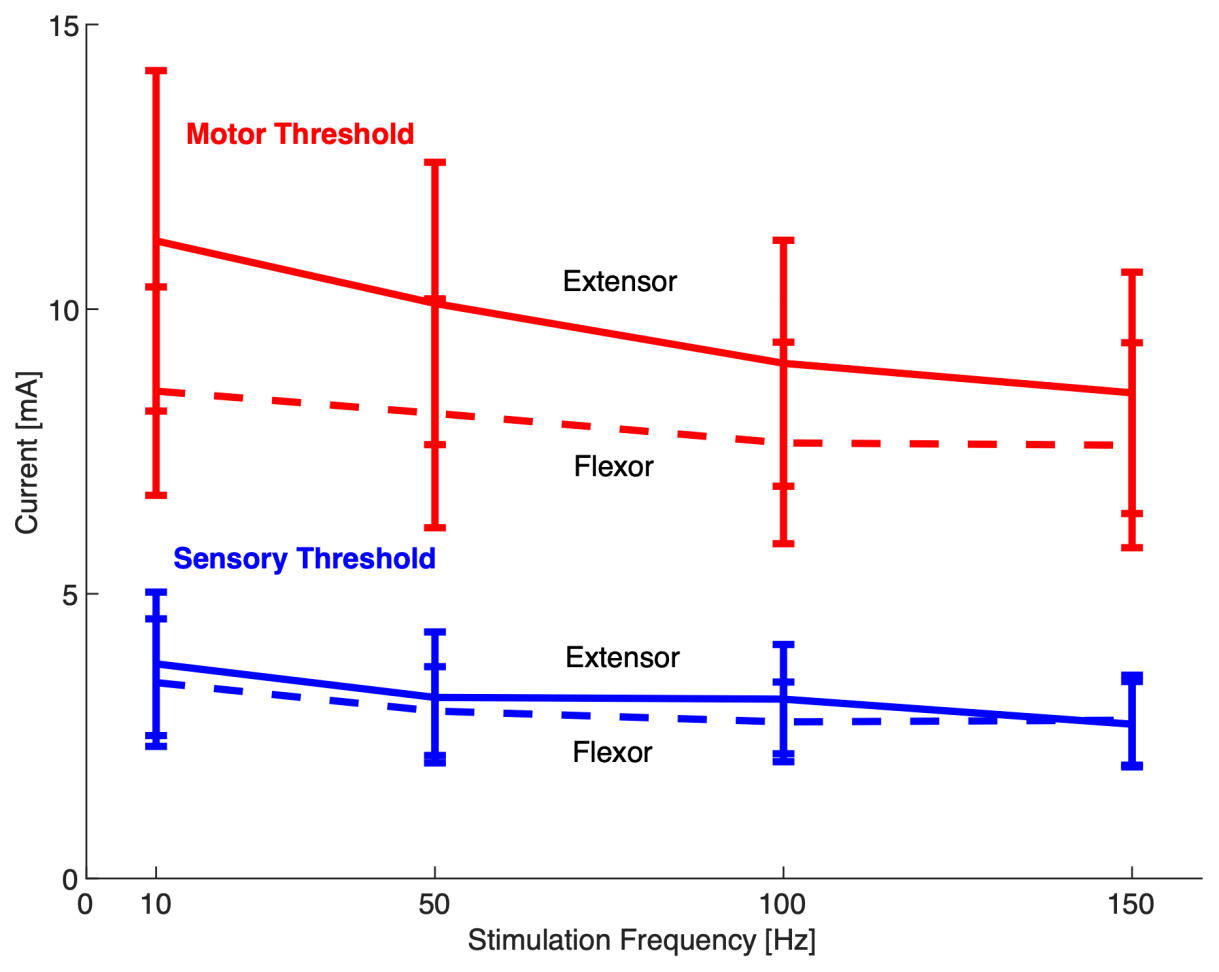
Average motor (red) and sensory (blue) thresholds of extensor carpi radialis (solid) and flexor carpi radialis (dashed). Bars indicate *±* 1 standard error.

### Experimental protocol

To test the effect of stimulation frequency on tremor, we performed 15 trials per subject, with a different stimulation frequency in each trial. The 15 stimulation frequencies included all frequencies between 10 and 150 Hz (the upper limit of the TENS device) in increments of 10 Hz (10 Hz, 20 Hz, …, 140 Hz, 150 Hz). These frequencies were tested in pseudo-random order, with a pulse width of 200 µs.

Each trial lasted 135s and consisted of three phases: 1) the BASE phase (30s) did not include any stimulation and was meant to measure the postural tremor baseline before stimulation; 2) the STIM phase (45s) involved continuous stimulation of the anterior and posterior sides of the forearm at one of the 15 frequencies; and 3) the REST phase (60s) did not include any stimulation to allow sensory receptors to return to resting state (see Limitations). Subjects were instructed to point the laser pointer at the target’s center in front of them for the entire trial. Two-minute breaks were taken in between every group of three trials, except for the last four trials, in between which there were no breaks. During each break, subjects were allowed to relax their wrist and remove their forearm from the support. The entire experiment, including setup, lasted approximately two hours.

### Data processing

To quantify the effects of stimulation on tremor, we calculated tremor power and frequency from both the sEMG and hand acceleration data.

#### Power

The sEMG recordings from the 4 muscles were processed in the same manner. The sEMG signals recorded during STIM phase were dominated by the stimulation and were therefore ignored. The signals from BASE and REST phases were filtered using a fourth-order Butterworth high-pass filter with a cutoff frequency of 20 Hz, full-wave rectified, and further processed in two ways. First, to visualize tremor power over time, we calculated spectrograms for each sEMG signal (using MATLAB’s pspectrum function, with type set to “spectrogram”, frequency limits ranging from 0 to 25 Hz, time resolution at 6 seconds, and all other inputs as default), resulting in separate power spectra for each 6-second interval. We integrated the spectrogram for each 6s interval across the tremor band (4–12 Hz) using the trapezoidal method, resulting in tremor-band power versus time. Second, to quantify differences in tremor power between phases, we returned to the high-pass filtered and rectified sEMG and calculated the power spectral density in the BASE and REST phases via MATLAB’s pwelch function using default values for the window, noverlap, and nfft inputs. The power spectral density was integrated (using the trapezoidal method) across the tremor band to yield a single measure of sEMG tremor-band power for both the BASE and REST phases for each muscle recorded.

To process the hand acceleration data, we detrended hand acceleration in each of the 3 axes of the hand-mounted accelerometer separately by subtracting mean acceleration values along each axis. The detrended data was also processed in two ways. First, to visualize tremor power over time, we calculated the spectrogram of detrended hand acceleration in all three phases (BASE, STIM, REST) along each acceleration axis using the same process and parameters described above for sEMG. The spectrograms along the three axes were added, and then integrated across the tremor band, resulting in hand-acceleration power in the tremor band over time. Second, to quantify differences in tremor power between phases, we returned to the detrended acceleration data, calculated the power spectral density along each of the axes (again using the same process and parameters as for sEMG), and integrated across the tremor band to yield power. The power values along the three axes were added together to yield a single measure of acceleration tremor-band power for each of the three phases.

#### Frequency

To determine the effect of stimulation on tremor frequency, we found the frequency of the peak in the tremor band of the power spectral densities of sEMG signals (only BASE and REST phases) and hand-acceleration (all three phases) for each trial. Peaks were identified using the sliding-window-constant-false-alarm-rate detection algorithm over 4–12 Hz [23], with 1.0 Hz sliding windows, 1.5 Hz sidebands, and a statistical significance of 5%. This algorithm categorizes data points in a sliding window that are statistically significantly greater than the data in the surrounding sidebands as a peak. The frequency of each peak was considered the tremor frequency.

### Statistical analysis

As a preliminary step, we estimated the effect of stimulation frequency on sensory and motor thresholds. Because a constant pulse width (200 µs) was used for all stimulation frequencies, the total amount of charge (area under the current vs time curve) was greater at higher stimulation frequencies. To quantify the effect of charge on sensory and motor threshold, we ran a mixed-model 2^nd^ degree factorial ANOVA of threshold data with the following factors: muscle (flexor, extensor), threshold type (sensory, motor), frequency (10, 50, 100, or 150 Hz), and subject (1–20), with subject as a random factor.

Our primary interest concerned on the effect of stimulation frequency on tremor power and frequency. Therefore, we performed mixed-model 2^nd^ degree factorial ANOVAs of both integrated tremor-band power and tremor frequency for the sEMG and hand acceleration data (four ANOVAs total). Neither integrated tremor-band power of sEMG nor hand acceleration were normally distributed, so they were log transformed before performing the ANOVAs. The following factors were included in each ANOVA: phase (BASE, STIM, and REST for hand acceleration; BASE and REST for sEMG), stimulation frequency (15 levels from 10 to 150 Hz), muscle (only for sEMG), sex, age, medication state (on/off), TETRAS score (to control for tremor severity), and subject (1–20), with subject as a random factor. A post-hoc analysis was performed using the Tukey Honest Significant Difference test.

In addition, we retrospectively analyzed the power associated with the interaction effect of phase and stimulation frequency (our effect of interest). Given the difficulties associated with mixed-model power analyses, this was performed using subject as a fixed factor.

## Results

### Sensory and motor thresholds

Subjects’ sensory and motor thresholds were measured in flexors and extensors at different frequencies (Figure 2). As expected, motor threshold was significantly greater than sensory threshold (p < 0.0001). Incidentally, the ANOVA also revealed that both sensory and motor thresholds were greater for extensors than flexors (p < 0.0001), and that stimulation frequency had a significant effect on thresholds (p < 0.0001) (Table 3). Specifically, the thresholds significantly decreased between 10 Hz and 100 Hz, between 50 Hz and 150 Hz, and between 10 Hz and 150 Hz, indicating that thresholds generally decreased slightly with higher stimulation frequencies.

**Table 3:** Effect of stimulation on motor and sensory thresholds.


SOURCE	*THRESHOLDS*			

	DF	DFDEN	F RATIO	PROB > F

Flexor vs. Extensor	1	272.1	32.867	<.0001*

Sensory vs. Motor Threshold	1	264.6	1051.2	<.0001*

Stimulation Frequency	3	268.2	11.230	<.0001*


### Effect of stimulation frequency on tremor power

**Figure 3:**
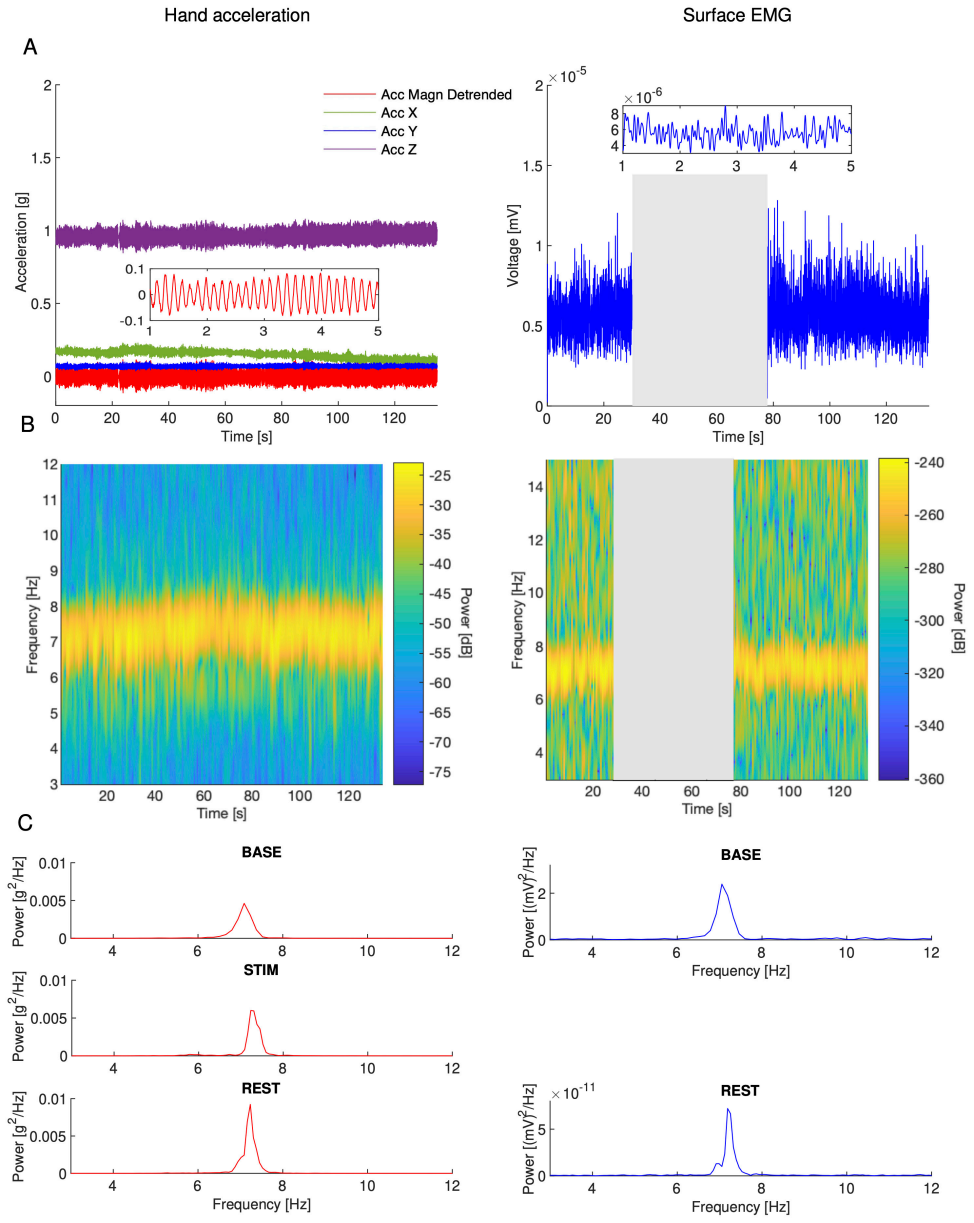
Data processing for a representative subject (subject 12 with 60 Hz stimulation) with no change in tremor after stimulation. **A.** Raw acceleration in each axis and the detrended magnitude of the acceleration vector (left) and the linear envelope of sEMG (right). The BASE phase (30s) was followed by the STIM phase (45s) and then the REST phase (60s). The STIM phase in the right subfigure is greyed out because the sEMG data was dominated by the stimulation. **B.** Spectrogram of acceleration (left) and processed sEMG (right). **C.** Power spectral density for acceleration (left) and for processed sEMG (right) for each phase.

**Figure 4:**
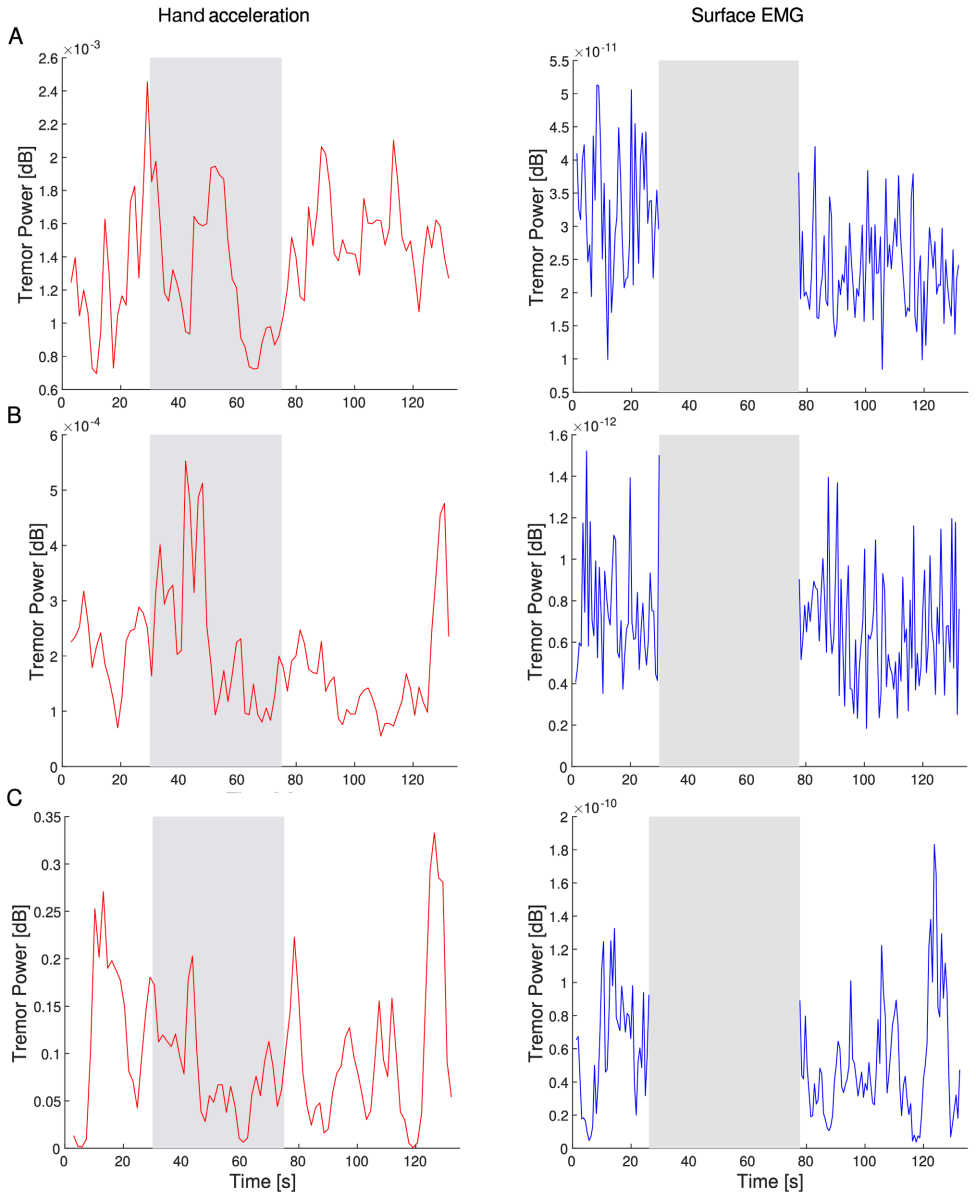
Hand acceleration power (left column) and sEMG power (right column) over time for three representative subjects and trials: Subject 12 with 60 Hz stimulation **(A)**, subject 10 with 120 Hz stimulation **(B)**, and Subject 11 with 60 Hz stimulation **(C)**.

Both hand acceleration and sEMG data were processed to yield spectrograms and power spectral densities (Figure 3). During the STIM phase, sEMG was dominated by stimulation and therefore ignored. Integrating the spectrograms across the tremor band yielded tremor-band power vs time (Figure 4). Tremor-band power vs time plots of individual trials showed a large diversity of behavior, including increases, decreases, plateaus, and seemingly random variation during all three phases.

**Figure 5:**
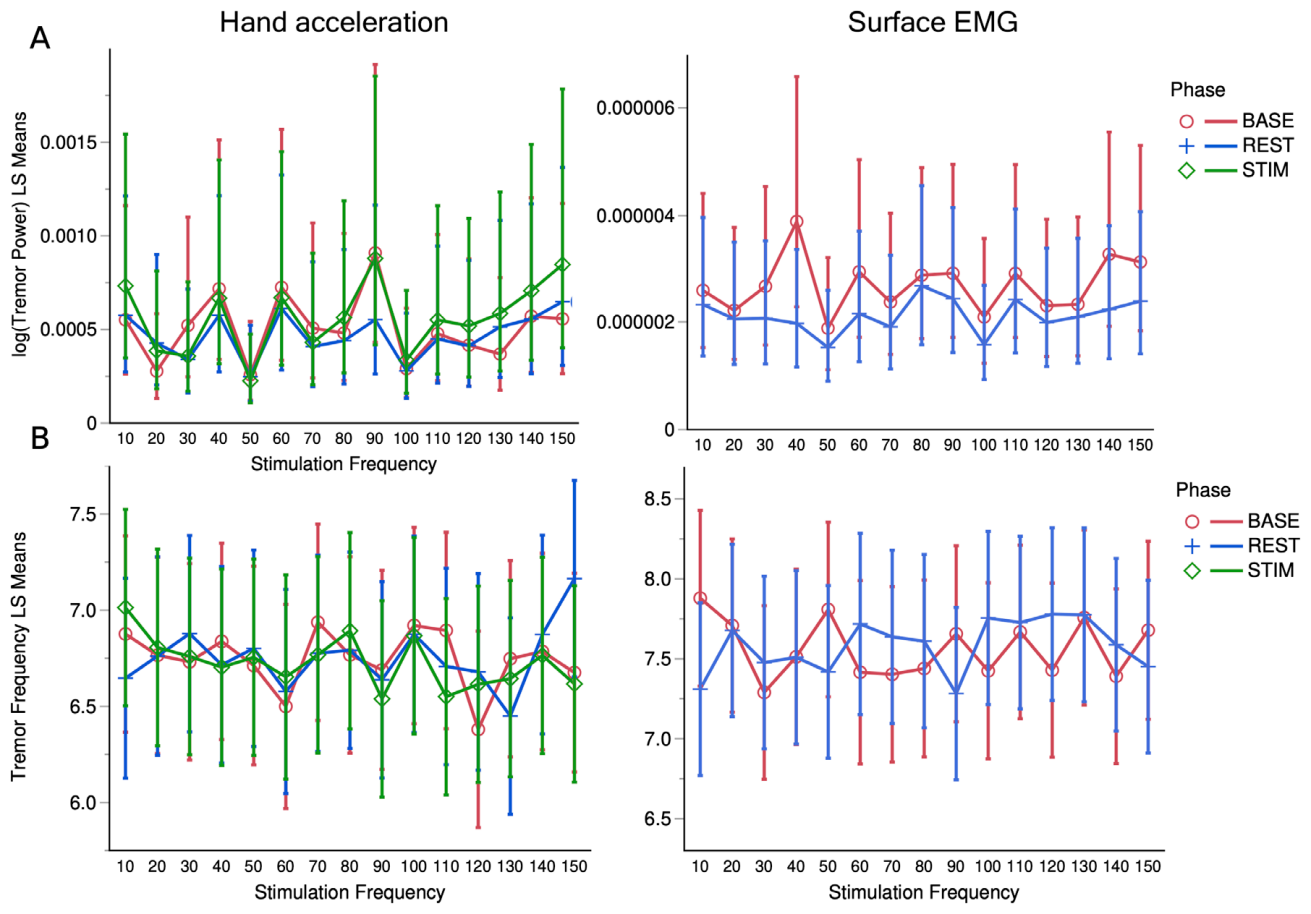
Least-squares means plots by phase (BASE, STIM, REST) for hand acceleration and sEMG data. **A.** Log-transformed power in acceleration (left) and sEMG (right). **B.** Peak frequency of acceleration (left) and sEMG (right). Bars indicate ±1 standard error.

**Figure 6:**
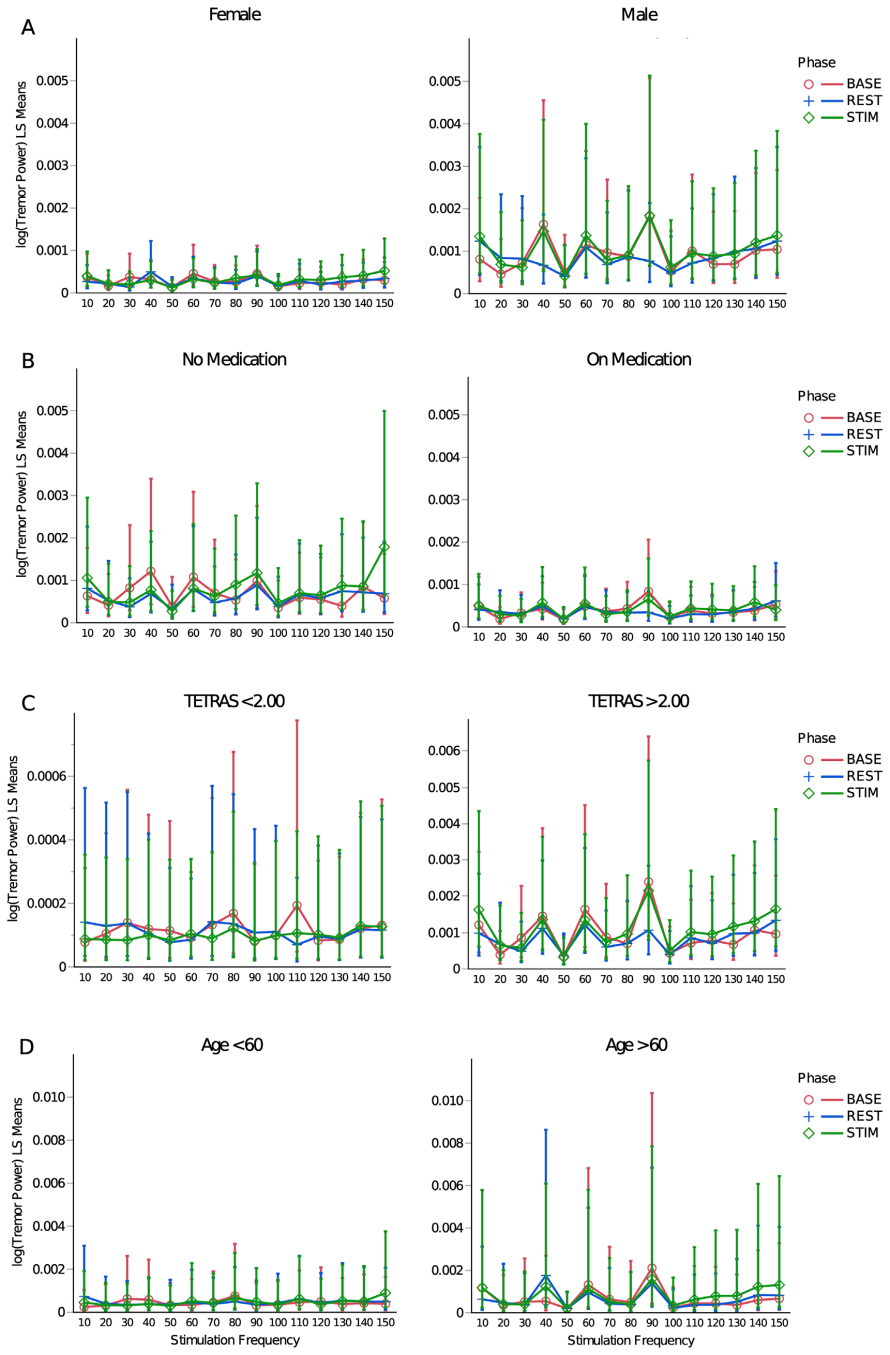
Least-squares means plots by phase (BASE, STIM, REST) for hand acceleration-derived tremor power, compared across control variable values, including sex **(A)**, medication state **(B)**, TETRAS score **(C)**, and age **(D)**. C. Note the difference in the scales of the vertical axes; TETRAS scores of 2, 3, and 4 are associated with mild, moderate, and severe tremor, respectively. Bars indicate ±1 standard error.

Combining results across all trials and subjects, our stimulation paradigm did not result in clinically significant tremor suppression—neither overall nor at any of the tested simulation frequencies (Figure 5). There was a statistically significant decrease in log-transformed tremor-band sEMG power from the BASE to the REST phase (p < 0.0001, Table 5), but the decrease was only 12.2 ±1.0%, and no corresponding decrease was observed in hand-acceleration power (p = 0.29, Table 4). Furthermore, there was no statistically significant interaction between phase and stimulation frequency for log-transformed tremor power measured by either hand acceleration (p = 0.69, Table 4) or sEMG (p = 0.07, Table 5). The p-value for sEMG (0.07) was close to significant, but a post-hoc Tukey HSD test revealed that this could be attributed to differences between unrelated trials (using different stimulation frequencies, rather than different phases of the same trial) and therefore carries little practical relevance. The retrospective fixed-model power analysis of the effect of phase interacted with stimulation frequency yielded a statistical power of 0.77 for the hand acceleration data and 0.89 for the sEMG data (at a significance level of 0.05 and given the model’s estimated error variance and effect size). Additionally, the interaction between phase and stimulation frequency for log-transformed tremor power measured by hand acceleration remained insignificant when split up by sex (p = 0.38), age (p = 0.99), medication use (p = 0.34), or TETRAS score (p = 0.96) (see Figure 6). For sEMG, the 3-way interaction of phase and stimulation frequency with sex (p = 0.006) and medication use (p = 0.045) were significant (Table 5). However, post-hoc Tukey HSD tests revealed that the significance of these 3-way interactions were attributable to differences between phases of unrelated trials involving different stimulation frequencies, thus carrying little practical significance. In summary, after extensive investigation, we did not find any other consistent patterns in log-transformed tremor power over time within any individual phase, within any of the control variables, or across the duration of the entire experiment.

**Table 4:** Stimulation effect on acceleration-derived tremor power and frequency in BASE, STIM, and REST.


SOURCE	*ACCELERATION-DERIVED LOG(TREMOR POWER)*				*ACCELERATION-DERIVED TREMOR FREQUENCY*			
	
	DF	DFDEN	F RATIO	PROB > F	DF	DFDEN	F RATIO	PROB > F

Stimulation Frequency	14	264	1.8853	0.0281*	14	264.8	0.4851	0.9401

Phase	2	30.29	1.2877	0.2906	2	30.76	0.1354	0.8738

Stimulation Frequency*Phase	28	416.4	0.8492	0.6900	28	414.4	0.8840	0.6394

Sex	1	14.99	4.9650	0.0416*	1	15.01	0.3178	0.5813

Age	1	15	2.6693	0.1231	1	15.02	6.0244	0.0268*

TETRAS Score	1	14.97	14.4631	0.0017*	1	15	5.1202	0.0389*

Medication	1	14.98	1.1456	0.3014	1	15.01	0.0162	0.9004

Sex*Phase	2	30.35	0.0019	0.9981	2	31.12	0.8142	0.4522

Age*Phase	2	30.73	0.2289	0.7968	2	31.38	0.0677	0.9347

TETRAS Score*Phase	2	30.02	0.6660	0.5212	2	31.06	1.5925	0.2196

Medication*Phase	2	30.25	0.1404	0.8696	2	31.04	0.2903	0.7501

Sex*Stimulation Frequency*Phase	28	416.5	1.0604	0.3842	28	414.8	1.1118	0.3195

Age*Stimulation Frequency*Phase	28	416.7	0.4141	0.9969	28	413.7	0.5557	0.9694

TETRAS Score*Stimulation Frequency*Phase	28	416.2	0.5848	0.9568	28	415.9	1.0143	0.4475

Medication*Stimulation Frequency*Phase	28	416.4	1.0978	0.3364	28	415.2	1.0083	0.4560


**Table 5:** Stimulation effect on sEMG-derived tremor power and frequency in BASE and REST.


SOURCE	*sEMG-DERIVED LOG(TREMOR POWER)*				*sEMG-DERIVED TREMOR FREQUENCY*			
	
	DF	DFDEN	F RATIO	PROB > F	DF	DFDEN	F RATIO	PROB > F

Stimulation Frequency	14	264	1.8081	0.0375*	14	265.1	0.3293	0.9899

Phase	1	15.08	31.9547	<.0001*	1	14.72	0.0290	0.8671

Muscle	3	56.99	27.4877	<.0001*	3	56.64	1.0141	0.3933

Stimulation Frequency*Phase	14	1893	1.6053	0.0705	14	1833	1.0849	0.3663

Stimulation Frequency*Muscle	42	1891	0.8859	0.6800	42	1831	0.8000	0.8171

Phase*Muscle	3	1891	56.9466	<.0001*	3	1834	2.1451	0.0926

Sex	1	15	1.0686	0.3176	1	15.01	1.9409	0.1839

Age	1	15.01	0.8733	0.3648	1	15.03	0.5542	0.4681

TETRAS Score	1	15	1.4125	0.2531	1	15.01	12.3494	0.0031*

Medication	1	15	0.0430	0.8385	1	15.01	0.0059	0.9398

Sex*Phase	1	15.12	5.5390	0.0325*	1	14.75	0.0006	0.9813

Age*Phase	1	15.41	2.8455	0.1118	1	15.02	0.6729	0.4249

TETRAS Score*Phase	1	14.87	0.0109	0.9181	1	14.76	0.0455	0.8340

Medication*Phase	1	15.05	1.9325	0.1847	1	14.77	0.2070	0.6557

Sex*Stimulation Frequency*Phase	14	1893	2.2029	0.0061*	14	1834	1.2260	0.2488

Age*Stimulation Frequency*Phase	14	1894	0.6494	0.8247	14	1836	1.2298	0.2460

TETRAS Score*Stimulation Frequency*Phase	14	1891	1.0095	0.4404	14	1839	1.4619	0.1175

Medication*Stimulation Frequency*Phase	14	1893	1.7275	0.0445*	14	1836	1.3433	0.1738


### Effect of stimulation frequency on tremor frequency

Our tremor peak detection algorithm successfully identified a tremor peak in the power spectrum for 97.1% of observations in the sEMG data and 99.4% of observations in the acceleration data.

Nevertheless, combining results across all trials and subjects, our stimulation paradigm did not result in significant tremor frequency changes – neither overall nor at any of the tested simulation frequencies (Figure 5). The effect of phase interacting with stimulation frequency on tremor frequency was statistically insignificant for the hand acceleration data (p = 0.64) and sEMG data (p = 0.37), even after controlling for sex, age, TETRAS score, and medication state (p > 0.11, Tables 4 and 5). The retrospective fixed-model power analysis of the same interaction effect yielded a statistical power of 0.79 for the hand acceleration data and 0.70 for the sEMG data (at a significance level of 0.05 and given the model’s estimated error variance and effect size). In addition, after extensive investigation, we did not find any consistent patterns in tremor frequency over time within any individual phase, within any of the control variables, or across the duration of the entire experiment.

## Discussion

The main purpose of this study was to test the effect of stimulation frequency on tremor suppression. Under our stimulation paradigm (45-second synchronous submotor stimulation over flexors and extensors), we did not find any evidence of clinically relevant tremor reductions nor tremor frequency changes at any stimulation frequency. The tremor-band sEMG power did decrease from the BASE to the REST phase, but the estimated effect was small (12%) and clinically irrelevant given that tremor power measured by hand acceleration (our main therapeutic target) remained unaffected. With statistical power values between 0.70 and 0.89, the retrospective power analysis confirmed that the results were not likely caused by insufficient sample size (type II error). Thus, we conclude that brief, synchronous submotor electrical stimulation over wrist flexor and extensor muscles is not effective in reducing or significantly changing tremor in patients with ET, independent of stimulation frequency (between 10 and 150 Hz).

While our results confirm those regarding tremor suppression using synchronous surface stimulation obtained by Pascual-Valdunciel et al., [8] who did not find any significant effect, they contrast with those obtained by Heo et al, [18] who found up to 90% tremor decrease. The reason for the differences in results obtained by Heo et al. (2015) are unknown, but we did note multiple methodological differences. Heo et al asked subjects to arrive at and then maintain a posture involving both arms stretched forward in front of the body. Although Heo et al. found significant tremor decreases measured by finger, hand, and wrist acceleration, they did not find any significant tremor decreases in the forearm. They also only used a 15s BASE phase, shorter than our 30s BASE phase (which was preceded by a 60s REST phase). Given that subject rested their arms by their sides and then made a large intentional movement to lift them during the beginning of the BASE phase of each new trial, 15s may not have been enough time to establish a true postural tremor baseline.

### Hypothesized Mechanisms underlying Tremor Suppression by Submotor Stimulation

To interpret our results and guide future research, we briefly review how they relate to recently published hypotheses explaining the effects of submotor stimulation on tremor. As mentioned in the introduction, the current literature contains sometimes contradictory results regarding synchronous submotor stimulation. In addition, the neural mechanisms underlying tremor suppression via submotor stimulation remain unclear. Still, two possible mechanisms connecting tremor suppression to the activation of afferent neurons by submotor stimulation emerge from the body of literature [8]. We will review each mechanism before discussing the accumulated supporting evidence and the implications of our results with regards to conducting future studies (see Table 6 for a summary of each hypothesized mechanism).

**Table 6:** Mechanistic hypotheses explaining how submotor peripheral stimulation could suppress tremor along with the required timing strategy and expected tremor effects.


HYPOTHESIS	MECHANISM OF TREMOR SUPPRESSION	STIMULATION TIMING REQUIRED FOR TREMOR SUPPRESSION	EXPECTED STIMULATION EFFECTS ON TREMOR

Supraspinal	Submotor stimulation disrupts central tremorgenic oscillators via afferent signal modulation	Irrelevant (synchronous or asynchronous)	Long-duration tremor suppression

			Possible tremor frequency changes

Spinal Circuit	Submotor stimulation partially cancels tremor signals via well-timed activation of reciprocal inhibition reflex	Only asynchronous	Instantaneous tremor suppression

			No tremor frequency changes


#### Supraspinal Hypothesis

Multiple authors have suggested that submotor peripheral stimulation disrupts supraspinal tremorogenic oscillations [8,16]. ET is driven by supraspinal pathological oscillatory activity in the central nervous system [24]. Peripheral stimulation activates afferent signals to the central nervous system, potentially altering the long-term dynamics of this pathological oscillatory activity and suppressing tremor. If this were the case, one could make three assumptions regarding different stimulation parameters. First, stimulation timing strategy (i.e., asynchronous versus synchronous) would likely be irrelevant as long as afferent signals induced by stimulation disrupt the central tremor network [14]. Second, tremor suppression effects would likely persist after stimulation ends due to the disruption of long-term tremorgenic dynamics [25]. Third, changing such long-term dynamics might result in tremor frequency shifts as seen in other tremor suppression experiments [21,26,27].

Several studies lend credibility to the supraspinal hypothesis. Some researchers found correlations between tremor suppression duration and tremor frequency, tremor frequency shifts after peripheral stimulation, and stimulation-dependent activation of the thalamic ventral intermediate nucleus (VIM), a component of the central tremor network and a target of tremor-suppressing DBS [21,25,28]. In addition, Pahwa et al. and Isaacson et al. performed submotor asynchronous stimulation on the medial and radial afferent nerves of ET patients for 40 minute stimulation durations, observing tremor reductions of up to 56% and for over 90 minutes after stimulation [15,17]. These observations all imply that peripheral submotor stimulation can affect central tremor networks and its tremor signals, as suggested by the supraspinal hypothesis.

Our results do not support or oppose the supraspinal tremor suppression hypothesis. Our sEMG measurements of tremor, the best proxy for descending input from supraspinal tremor networks [21,25], showed only small changes in tremor power that failed to translate into clinically relevant tremor reductions (as measured by hand acceleration). However, disruptions to supraspinal tremor generation might require stimulation durations significantly longer than ours. Our small but statistically significant sEMG tremor-band power reduction might represent the beginning of supraspinal effects, possibly requiring longer stimulation on the order of 40 minutes as seen in Pahwa et al. and Isaacson et al. [15,17] Given the simplicity of implementing synchronous stimulation, we suggest that future studies test the effects of long-duration (ca. 40 minutes) synchronous submotor stimulation on ET power and frequency to evaluate the merits of this hypothesis.

#### Spinal Circuit Hypothesis

According to the second hypothesized mechanism, an asynchronous, out-of-phase, submotor stimulation strategy might counteract tremor-related contractions via reciprocal inhibition. Reciprocal inhibition occurs when Ia-afferent neurons activate the muscle from which they originate but also disynaptically inhibit the neural drive to antagonist muscles via spinal reflex circuitry [29]. By measuring tremor frequency in real time and timing peripheral submotor stimulation just right, one could activate Ia-afferents so that this reciprocal inhibition reflex inhibits the antagonist muscle just as tremor bursts arrive [30]. Under this mechanism, “the appropriate timing of stimulation could be critical” [14]. Synchronous stimulation would not suppress tremor, but asynchronous stimulation would. In addition, we would not expect tremor frequency shifts or long-term tremor suppression since supraspinal tremor networks remain unaffected.

Previous studies using asynchronous submotor stimulation have shown instantaneous tremor reductions, lending support to the spinal circuit hypothesis. Using an asynchronous submotor stimulation paradigm, Dosen et al. and Dideriksen et al. observed tremor suppression effects of up to 42% only “while the stimulation was being delivered”, as predicted by the spinal circuit hypothesis [9,14]. In addition, Dideriksen et al. showed that intramuscular stimulation of proprioceptive Ia afferents was more effective than surface stimulation, as would be expected if the spinal circuit hypothesis is true, given that reciprocal inhibition is mediated by Ia afferents.

The results of our study are in harmony with the spinal circuit hypothesis. The spinal circuit hypothesis predicts that brief *synchronous* stimulation should have no effect since simultaneous reciprocal inhibition from antagonist muscles, such as the wrist flexors and extensors we stimulated, would cancel out. This is consistent with our results since we saw no clinically relevant tremor power or frequency changes under our stimulation paradigm. This is also consistent with the results obtained by Pascual-Valdunciel et al., [20] while contrasting with those obtained by Heo et al. [18] Future studies could evaluate this hypothesis further by measuring tremor signals to antagonist muscles during asynchronous stimulation, although this may prove technically challenging.

### The effect of stimulation on sensory and motor thresholds

Although the main purpose of measuring sensory and motor thresholds was to conduct a consistent submotor-threshold stimulation protocol at different stimulation frequencies, it is still interesting to note that sensory and motor thresholds (in terms of electrical current needed to activate sensory and motor neurons) decreased with higher stimulation frequency. We expected this since higher stimulation frequencies imply a higher total charge (integrated sum of electrical current) being applied to the skin surface during stimulation. That said, we would have expected thresholds to be inversely proportional to stimulation frequency (to maintain a given charge), but the observed decrease in thresholds was significantly less than that (Figure 2).

### Limitations

Our study included several limitations. Our sample of 20 subjects was small and heterogenous, although we controlled for several factors (sex, age, TETRAS score, and medication state). According to their TETRAS scores, the majority of subjects had mild-to-moderate tremor, with only one subject exhibiting moderate-to-severe tremor (Table 2). We only used postural tasks to evaluate tremor and excluded tasks related to kinetic tremor. Also, we did not perform repetitions of trials for any frequencies or patients, which would have increased the sample size. That said, according to our retrospective power analysis, our sample size was sufficiently large to yield power levels of 0.77 and 0.89 for tremor power and 0.70 and 0.79 for tremor frequency, which are close to the standard of 0.80.

Although we stimulated at different currents for different stimulation frequencies (to keep the stimulation halfway between sensory and motor thresholds), we kept the pulse width constant at 200 µs, independent of stimulation frequency. The effect of pulse width on tremor suppression is unknown. The total charge output by the stimulation device increases linearly with pulse width and stimulation frequency, but the effect of total charge on tremor suppression is also unknown (as mentioned above and shown in Figure 2, sensory and motor thresholds do not depend linearly on charge). Furthermore, as listed in Table 1, past tremor-suppression studies using submotor threshold stimulation have used pulse widths of either 300 or 400 µs (stimulating at 100 Hz) or 650 µs (stimulating at 150 Hz), without any observable pattern in the efficacy of tremor suppression. Instead, efficacy of tremor suppression appears to be correlated with agonist-antagonist timing (synchronous vs asynchronous) and duration (brief- vs long-duration), as explained above.

As mentioned above, we were unable to detect motor threshold for 8 subjects and used the maximum tolerable current rather than the stimulation current between motor and stimulation threshold. For the investigation of the effect of stimulation frequency on sensory and motor thresholds, our previous analysis already excluded missing observations. For the investigation of the effect of stimulation frequency on tremor power and frequency, the missing observations may have induced bias into our statistical analysis. Therefore, we repeated the analyses described above, but excluding those 8 subjects. We did not see any statistically significant interaction between phase and stimulation frequency for tremor power measured by either hand acceleration (p = 0.47) or sEMG (p = 0.57). The effect of phase interacting with stimulation frequency on tremor frequency was also statistically insignificant for the hand acceleration data (p = 0.49) and sEMG data (p = 0.23). This mirrors our previous results and lends further robustness to our previous conclusions.

Although the 1” × 1” electrodes used in this study are smaller than those used in many prior studies [14,18,19,20], they did not allow precise control over exactly what was stimulated. That said, during the calibration phase, we increased the current until it produced muscle contraction (in all but the 8 subjects mentioned above), indicating that muscles were stimulated. We then decreased the current to halfway between sensory and motor thresholds, which corresponded to about 2/3 of motor threshold (Figure 2). In other words, during the experiment, each subject (except for the eight subjects mentioned above) was stimulated at roughly 2/3 the current known to cause muscle contraction in that subject.

Another limitation is the length of time between each trial. Our protocol called for a rest phase of 60s followed by a baseline phase of 30s before applying stimulation at a different frequency. We were unable to find measured estimates of the refractory period of muscle fibers when using submotor-threshold TENS, so we chose the durations of the BASE and REST phases based on research relating to vibrational stimulation, which suggested that a length of 40–60s was sufficient for muscle spindle recovery [31]. In our thorough investigation, we did not find that the BASE phase of one trial was affected by previous trials, so a rest phase of 60s may have been sufficient, but it is difficult to state this with confidence without further experimentation.

## Conclusions

We conclude that brief, synchronous submotor stimulation over peripheral muscles is not effective in reducing postural tremor or changing tremor frequency, independent of stimulation frequency (in the range of 10–150 Hz). In conjunction with studies that suppress tremor using asynchronous submotor stimulation, our results are congruent with the spinal circuit hypothesis of tremor suppression via reciprocal inhibition. Due to our brief 45s stimulation duration, we cannot make conclusions regarding the supraspinal hypothesis of tremor suppression via central tremor network disruption.

For future study, we suggest investigating the effects of combining synchronous submotor stimulation with long stimulation durations like Pahwa et al. and Isaacson et al [15,17]. According to the supraspinal hypothesis, this should lead to long-term tremor suppression and might shift tremor frequency. Under such a long-term synchronous submotor stimulation paradigm, we could also compare intramuscular stimulation (which directly targets proprioceptive Ia afferents) to surface stimulation (targeting cutaneous sensory afferents) to tease out pathways conducting disruptive signals to the central tremor network. To our knowledge no such study has been attempted, despite potentially solidifying the mechanistic explanations for long-term tremor suppression.

Additionally, the spinal circuit hypothesis can be explored further using different stimulation parameters under an asynchronous stimulation timing strategy. To our knowledge, no study has evaluated the effect of different stimulation frequencies on tremor suppression using asynchronous submotor stimulation. Other parameters like pulse width, stimulation duration, and stimulation site also require further investigation to optimize brief, asynchronous submotor stimulation for future therapeutic applications.
